# Ceftazidime–avibactam resistance in *Klebsiella pneumoniae* sequence type 37: a decade of persistence and concealed evolution

**DOI:** 10.1099/mgen.0.000931

**Published:** 2023-02-08

**Authors:** Gabriele Arcari, Riccardo Polani, Francesco Bruno, Valerio Capitani, Federica Sacco, Gaia Menichincheri, Giammarco Raponi, Alessandra Carattoli

**Affiliations:** ^1^​ Department of Molecular Medicine, Sapienza University of Rome, Rome, Italy; ^2^​ Department of Public Health, Sapienza University of Rome, Rome, Italy; ^3^​ Department of Molecular Medicine, Sapienza University of Rome, Rome, Laboratory affiliated to Istituto Pasteur Italia - Fondazione Cenci Bolognetti, Italy

**Keywords:** endemicity, KPC, pKpQIL, ST_37_

## Abstract

The first reports of carbapenem-resistant *Enterobacterales* in our hospital date back to 2006. In that period, few ertapenem-resistant but meropenem-susceptible *Klebsiella pneumoniae* isolates belonging to sequence type (ST) 37 were retrieved from clinical samples. These strains produced the CTX-M-15 extended spectrum β-lactamase, OmpK35 was depleted due to a nonsense mutation, and a novel OmpK36 variant was identified. Yet, starting from 2010, *Klebsiella pneumoniae* carbapenemase (KPC)-producing ST512 isolates started prevailing and ST37 vanished from sight. Since 2018 the clinical use of the combination of ceftazidime–avibactam (CZA) has been introduced in clinical practice for the treatment of bacteria producing serine-β-lactamases, but KPC-producing, CZA-resistant *K. pneumoniae* are emerging. In 2021, four CZA-resistant ST37 isolates producing KPC variants were isolated from the same number of patients. *bla*KPC gene cloning in *Escherichia coli* was used to define the role of those KPC variants on CZA resistance, and whole genome sequencing was performed on these isolates and on three ST37 historical isolates from 2011. CZA resistance was due to mutations in the *bla*KPC genes carried on related pKpQIL-type plasmids, and three variants of the KPC enzyme have been identified in the four ST37 strains. The four ST37 isolates were closely related to each other and to the historical isolates, suggesting that ST37 survived without notice in our hospital for 10 years, waiting to re-emerge as a CZA-resistant *K. pneumoniae* clone. The ancestor of these contemporary isolates derives from ST37 wild-type porin strains, with no other mutations in chromosomal genes involved in conferring antibiotic resistance (*parC*, *gyrA*, *ramR*, *mgrB*, *pmrB*).

## Data Summary

1. A BioProject has been released at DDBJ/ENA/GenBank, no.: PRJNA853564 (https://dataview.ncbi.nlm.nih.gov/object/PRJNA853564).
2. Circular complete genome plasmids and phages of 1020, 1021, 4011 and 9362 strains have been released under accession nos. CP100312–CP100315, CP100308–CP100311, CP101896–CP101902 and CP101890–CP101895, respectively.
3. The *bla*_KPC-110_ allele in pKpQIL_1020 from strain 1020 has been released under NCBI accession no. CP100313.
4. pKpQIL_17B plasmid from strain ST512 17B is released under NCBI accession. no. MT809697.

**Impact Statement**

Owing to its capacity to collect multiple antimicrobial-resistance determinants, *Klebsiella pneumoniae* is one of the greatest threats to public health. Typically, the dissemination of multidrug-resistant *K. pneumoniae* is driven by a few high-risk clones, such as those belonging to clonal groups (CGs) 101, 147, 258 and 307. Even though *K. pneumoniae* isolates belonging to sequence type (ST) 37 have been listed as a high-risk multidrug-resistant clone, typically linked to the dissemination of the 16S rRNA methylases RmtB and ArmA, this ST is poorly represented in both genomic databases and the scientific literature. In this study, we dissected an outbreak of ceftazidime–avibactam-resistant isolates belonging to ST37, comparing their genomes with others from different times and places belonging to the same ST. ST37 was the endemic clone in our hospital 10 years ago, when it was overshadowed by *K. pneumoniae* carbapenemase (KPC)-producing CG258 isolates. Today, ST37 is striking back, with four KPC-producing isolates resistant to ceftazidime–avibactam.

## Introduction

Carbapenem-resistant *Enterobacterales* are one of the greatest threats to public health systems worldwide [1]. In Italy, the European Centre for Disease Prevention and Control surveillance system reports dramatic increases of carbapenem-resistant *Klebsiella pneumoniae* from 1 % in 2009 to 34 % in 2016 (currently, 2020, 29.5 %; https://atlas.ecdc.europa.eu/). In Italy, the main representative of this threat is *K. pneumoniae* carbapenemase (KPC)-producing isolates, especially the sequence types (STs) 512, 307 and 101 [2]. The resistance to carbapenems is the outcome of decades of evolution, mainly based on horizontal gene transfer of plasmids carrying multiple resistance genes [3, 4].

In the Policlinico Umberto I (PUI) hospital, carbapenem resistance in Gram-negative bacteria was not reported until 2006. However, a few ertapenem-resistant, meropenem-susceptible *K. pneumoniae* strains were identified at that time [5], which were assigned to ST37 based on multi-locus sequence typing (MLST [6]). These strains carried the genes coding for the CTX-M-15 extended spectrum β-lactamase (ESBL) and showed the depletion of OmpK35 due to a nonsense mutation, associated with a novel OmpK36 variant [5]. Resistance to meropenem was observed in the ESBL-producing ST37 strains that did not produce either the OmpK35 or the OmpK36 porin [5].

Carbapenem-resistant ST37 strains were not reported after the introduction in the PUI hospital of isolates belonging to clonal group (CG) 258 carrying *bla*_KPC_. ST37 *K. pneumoniae* seemingly disappeared, substituted by KPC-producing clones [7].

A decade later, the spread of carbapenem resistance in *K. pneumoniae* had not ceased. Since 2018 the clinical use of the combination of ceftazidime–avibactam (CZA) has been introduced in the hospital for treatment of bacteria producing serine-β-lactamases. Yet, KPC-producing CZA-resistant *K. pneumoniae* strains are emerging [8, 9].

Here, starting from the report of four KPC-producing CZA-resistant ST37 *K. pneumoniae* isolated in 2021 causing respiratory tract and bloodstream infections, we endeavour to reconstruct the evolution of this clone at PUI and compare contemporary and historical isolates.

## Methods

### Bacterial strain isolation and susceptibility testing

Isolated colonies were identified by a MALDI-TOF MS system (Bruker Daltonik). Antimicrobial susceptibility was determined by the MicroScan WalkAway system (Beckman Coulter). Isolates showing a carbapenem-resistant phenotype were tested using the real-time PCR assay Xpert Carba-R kit for the GeneXpert system (Cepheid). CZA minimal inhibitory concentrations (MICs) were determined using the CZA gradient test (Lioﬁlchem). CZA-resistant isolates carrying *bla*_KPC_ were subjected to complete DNA sequencing of the *bla*_KPC_ gene obtained by amplification, as previously described [10].

### Whole genome sequencing and assembly

Whole genome sequencing (WGS) was obtained by Illumina MiSeq (Illumina) after genomic DNA purification following the Isolate II genomic DNA Extraction Kit procedure (Bioline) for four contemporary and three historical ST37 isolates. The Nextera XT DNA sample preparation kit generated paired-end libraries with the 2×300 paired-end protocol (Illumina). Illumina reads were assembled at the public Europe Galaxy Server (https://usegalaxy.eu/) by version 3.15.3 of the SPAdes [11] pipeline.

Representative isolates of contemporary and historical isolates were also subjected to Oxford Nanopore Technologies (ONT) sequencing, as previously described [10].

Illumina reads and ONT assemblies were integrated by the Unicycler tool version 0.4.8.0 using a bold bridging mode [12].

### Phylogenesis and synteny

Thirty-nine genome sequences (seven obtained in this study and 32 downloaded from the GenBank database) were annotated using Prokka version 1.14.6 [13] and the resulting general feature formats (GFFs) were analysed using Roary v3.11.3 [14] to build a core genome alignment and a gene presence/absence tabular file.

A consensus phylogenetic tree based on 100 000 bootstraps was generated with IQ-TREE using the TIM+F+I model of substitution [15]. The tree and metadata were visualized with Microreact [16] and adjusted using the open-source InkScape software.

The temporal signal resulting from these phylogenetic data was evaluated by TempEst v1.5.3 using a root-to-tip regression analysis as a function of the sampling time [17]. This preliminary analysis yielded a low correlation coefficient (0.1871 best root), and thus no Bayesian-based analysis was performed to infer a time-scaled phylogeny for these isolates.

Synteny maps were created by blastn and visualized using the Circos tool [18], adjusting the resulting plot with the open-source InkScape software.

### Analysis of the core and of the accessory genome

Single Nucleotide Polymorphisms (SNPs) were analysed by the Snippy tool (https://github.com/tseemann/snippy) to determine the number and the position of the SNPs between isolates 9362 and 1020. Given the lack of core-genome distance between contemporary isolates, isolate 1020 was arbitrarily chosen as the reference. All chromosomal SNPs were plotted over the 1020 genome. Furthermore, we performed a Clustal Ω alignment [19] on proteins encoded by specific chromosomal genes associated with antimicrobial resistance (*gyrA* and *parC* for fluoroquinolone resistance, *ramR* for tigecycline resistance, *ompK35* and *ompK36* for β-lactam resistance, *mgrB* and *pmrB* for colistin resistance). Capsular polysaccharide (CPS) and the lipopolysaccharide (LPS) loci were analysed using the Kaptive tool [20].

To evaluate which genes were present in the contemporary isolates and which were present in the historical ones, we deployed the Scoary tool [21]. In addition, we performed a function- and a sequence-based analysis by the Rapid Annotation of microbial genomes using Subsystems Technology (RAST) and the associated SEED tools [22]. Major genomic differences, defined by the presence or absence of at least five consecutive coding sequences (CDSs), were manually curated.

We also analysed the virulence content using Kleborate [23], the presence of CRISPR loci using CRISPR-Cas finder [24] and of phages using PHASTER [25] tools.

### pKpQIL plasmid transformation

Plasmid DNA was extracted from overnight growth of 10 ml liquid LB broth of *K. pneumoniae* isolate 1020 by the PureYield plasmid midiprep system (Promega Italia). Purified plasmid DNA was used to transform chemically competent *Escherichia coli* DH5-α cells (Life Technologies, Thermo Fisher Scientiﬁc), selecting transformants on LB agar plates containing ceftazidime (6 mg l^−1^). After 24 h, colonies were screened for *bla*_KPC_ and replicon content (PBRT 2.0; Diatheva). *E. coli* DH5-α transformants positive for both *bla*_KPC_ and the FIB replicon specific for the pKpQIL plasmid were tested in triplicate for several antibiotics by microdilution (MicroScan system). CZA MICs were tested in triplicate by the CZA gradient test (Lioﬁlchem).

### Cloning of *bla*_KPC_ mutants in pCR-Blunt II TOPO vector

Each isolate carrying a different *bla*_KPC_ mutant was used as template for an AccuPrime Pfx DNA polymerase-based PCR as previously described [10] and ligated into the pCR-Blunt II TOPO vector (ThermoFisher). Ligation mixtures were introduced by transformation in chemically competent TOP10 cells (Life Technologies, Thermo Fisher Scientiﬁc) and transformants were screened on LB agar plates containing kanamycin (ImMedia Kan agar; Invitrogen, Thermo Fisher Scientiﬁc). The accuracy of the cloning was assessed by PCR and Sanger sequencing of the amplicons as previously described [10]. *E. coli* TOP10 cells producing KPC variants were tested in triplicate for several antibiotics by microdilution (MicroScan system). CZA MICs were tested in triplicate by the CZA gradient test (Lioﬁlchem).

## Results and discussion

### *Klebsiella pneumoniae* ST37 collection

In this study, seven *K. pneumoniae* isolates were studied and WGS was performed. Strains from the historical collection (SC26, 9362 and 4011) were isolated in 2011 during a KPC-positive and KPC-negative case-control study performed in different hospitals of Rome, Italy [26]. Among the KPC-negative isolates, ST37 9362 (ertapenem-susceptible) and 4011 (ertapenem-resistant) strains were identified at the PUI, while isolate SC26 (ertapenem-resistant) was from the San Camillo Hospital.

Bacterial isolates from the contemporary collection (1015, 1016, 1020, 1021) were isolated from samples collected during routine microbiological processes in PUI hospital in 2021. The four bacterial strains were from four different infected patients hospitalized in the same ward, causing respiratory tract infections or bacteraemia (Table 1).

**Table 1.** Genetic characteristics identified in *Klebsiella pneumoniae* ST37 strains isolated in 2011–2021, Rome, Italy

Isolate	Year	Source	Beta-lactamase genes	Other AMR genes	Chromosomal mutations conferring AMR	Plasmid content
1015	2021	Respiratory tract	*bla*_KPC-70_, *bla*_OXA-9_, *bla*_TEM-1A_	ND	WT	pKpQIL, pKPN
1016	2021	Respiratory tract	*bla*_KPC-31_, *bla*_OXA-9_, *bla*_TEM-1A_	ND	WT	pKpQIL, pKPN
1020	2021	Blood culture	*bla*_KPC-110_, *bla*_OXA-9_, *bla*_TEM-1A_	ND	WT	pKpQIL, pKPN
1021	2021	Blood culture	*bla*_KPC-31_, *bla*_OXA-9_, *bla*_TEM-1A_	ND	WT	pKpQIL, pKPN
SC26	2011	Respiratory tract	*bla*_CTX-M-15_, *bla*_OXA-1_, *bla*_OXA-9_, *bla*_TEM-163_	*aac(3)-IIa, aac(6')-Ib-cr, aadA1, aph(3'')-Ib, aph(6)-Id, armA, catA1*, *catB4*, *dfrA5*, *mph(E), msr(E*), *sul1*, *sul2*, *tet(D*)	ParC K84E, OmpK35Y36X, OmpK36 135_136insDT	IncF, pKPN, IncR
9362	2011	Blood culture	*bla*_CTX-M-15_ *, bla*_OXA-1_ *, bla*_TEM-1B_	*aac(3)-IIa, aac(6')-Ib-cr, armA, catA1, catB4, dfrA5, mph(E), msr(E), sul1, tet(D*)	ParC K84E, OmpK35Y36X	IncF, pKPN, ColRNAI
4011	2011	Urine	*bla*_CTX-M-15_ *, bla*_OXA-1_	*aac(3)-IIa, aac(6')-Ib-cr, catA1, catB4, dfrA5, sul1*	ParC K84E, OmpK35Y36X, OmpK36 135_136insDT	IncF, pKPN, ColRNAI

AMR, antimicrobial resistance.

ND, not detected

WT, wild type

### Retrieval of ST37 isolates from the GenBank database

As of June 2022, 2934 completely assembled *K. pneumoniae* genomes were downloaded from the public NCBI dataset (https://www.ncbi.nlm.nih.gov/datasets/docs/v1/how-tos/genomes/download-genome/) and screened by the Kleborate [23] and the Kaptive [20] tools. Thirty-two univocal isolates belonging to ST37 were retrieved and included in the study for genomic comparison.

### Analysis of the core genome in contemporary and historical ST37

Thirty-nine genome sequences (seven obtained in this study and 32 downloaded from the GenBank database) were used to build a core genome-based alignment.

**Fig. 1.**
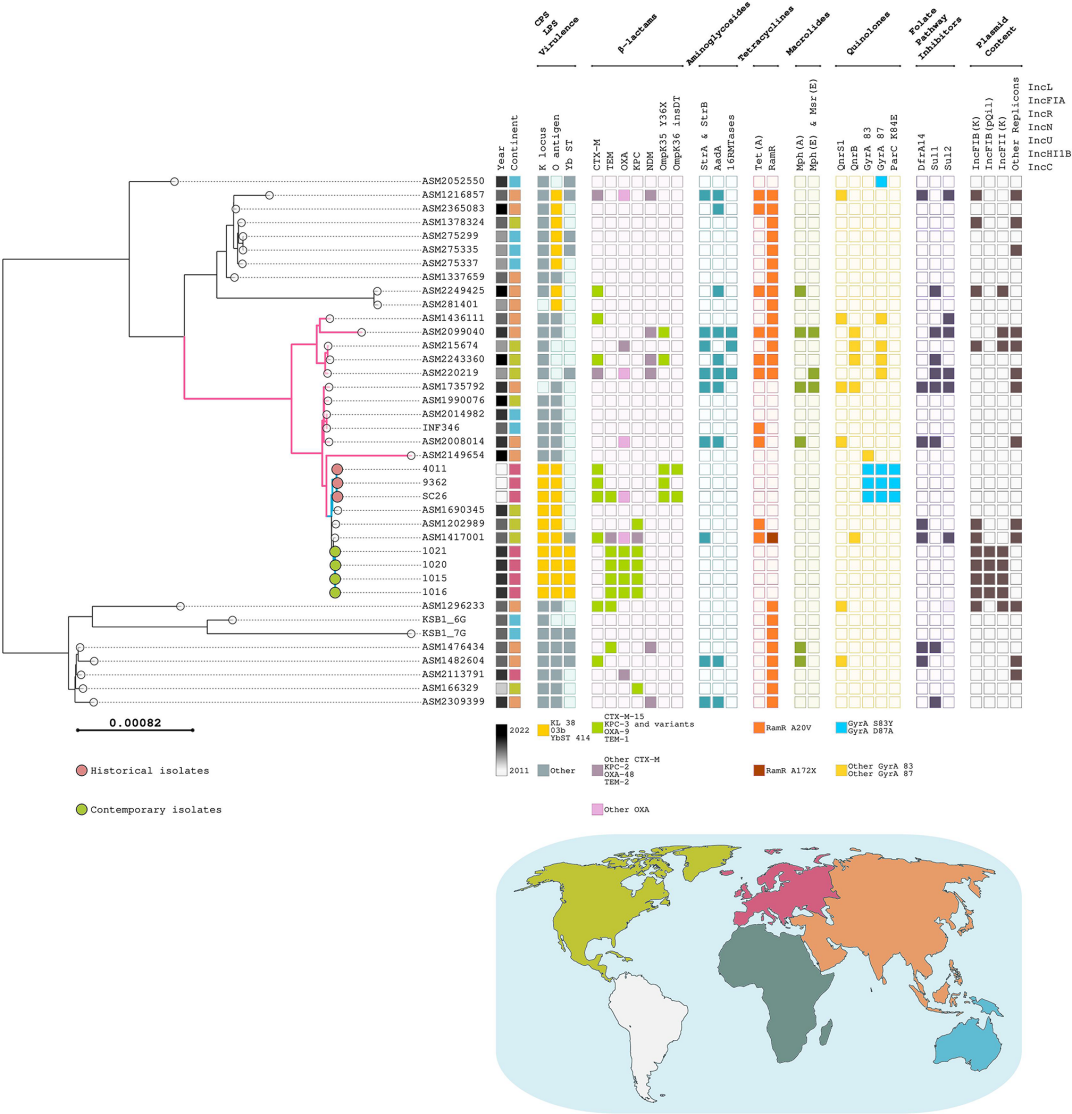
Phylogenetic analysis of *Klebsiella pneumoniae* ST37. Microreact visualization adjusted using the open-source InkScape software of a midpoint-rooted phylogenetic tree of the 39 ST37 genomes (32 retrieved from the GenBank database and seven ST37 from this study) built using IQ-TREE. Isolation year is colour-coded according to a black–white gradient; isolation continent is colour-coded according to the world map beneath the tree. Empty squares indicate that the specific feature has not been identified in the genome or, in the case of OmpK35, OmpK36, GyrA, ParC and RamR, that the protein is wild-type. OmpK36 insDT indicates the DT insertion between amino acid positions 135 and 136; OmpK35Y36X indicates the presence of a stop codon in the position corresponding to amino acid position 36. Coloured squares indicate the presence of a specific feature identified in the genome, listed in the key below the respective metadata columns.

The ST37 phylogenetic tree shows two deep branches (Fig. 1). One less populated branch (eight genomes) appears to be rather heterogeneous, while the most populated one (31 genomes) is made up of three major branches. The historical and the contemporary ST37 isolates from Rome displayed a rather conserved core genome and were in one of these three branches (coloured in pink in Fig. 1), sharing a tight phylogenetic bond (coloured in blue in Fig. 1). On this branch, three genomes isolated from patients in the USA (New York, ASM1417001; Chicago, ASM1202989; Washington, ASM1690345) demonstrated close phylogenetic relationships with historical and contemporary ST37 isolates from Italy.

Chromosomal differences were calculated between isolates 9362 and 1020 as representatives of the historical and contemporary collections, respectively. This analysis identified 470 total SNPs accumulated in the decade separating the two compared strains, corresponding to an average of 47 mutations/genome per year (Dataset SD1 available in the online version of this paper). As a term of comparison, we used the previously described *K. pneumoniae* evolutionary rate calculated as being 10.1 substitutions/genome/year [27]. Therefore, the accumulation of mutations in ST37 is observed to be higher, despite the fact that estimation of the mutations gained in short timeframes may vary according to several factors (i.e. mutation rate, generation time and natural selection), greatly magnifying, or reducing, the evaluation of this evolutionary measure [28].

### Comparison of capsule and other chromosomal genes

In *K. pneumoniae,* the diversity of LPS antigens is currently restricted to 13 different serotypes. Regarding CPS the situation is more complex, since there are 77 serologically defined K-types [20, 29]. *In silico* analysis [20] revealed that contemporary and historical isolates from Rome shared the same outer LPS (O3b) and CPS (KL38) antigens. Analysing the 32 retrieved genomes, O3b LPS antigen was identified in nine other ST37 genomes. By contrast, KL38 was detected only in the three above-mentioned genomes from the USA (Fig. 1).

There is no co-linear evolution between historical and contemporary isolates considering chromosomal genes linked to antimicrobial resistance. While the three historical isolates presented a premature stop codon at position 36 in the outer membrane protein OmpK35, the contemporary isolates expressed the wild-type porin (9 % coverage because of the frameshift, 100 % identity in the 35 translated amino acids). Furthermore, OmpK36 in ertapenem-resistant SC26 and 4011 strains presented a DT insertion at position 135-136 compared to the wild-type porin, identified in the ertapenem-susceptible 9362 genome (100 % coverage and 99.42 % identity in the amino acid sequence). These OmpK36 variants were previously described as associated with ertapenem resistance [5] and were not found in contemporary isolates. This observation suggests that the ancestor of the contemporary KPC-producing isolates identified at PUI came from an ertapenem-susceptible, wild-type porin strain. Our hypothesis is that the ST37 clone which has been able to survive up to the acquisition of *bla*_KPC_ was the one with fewer mutations, and not the most resistant one. In fact, despite the outer membrane protein alterations conferring higher MICs for carbapenems, they have a cost to bacterial fitness [30].

This line of reasoning can also be applied to the novel variant (K84E) of ParC (100 % coverage and 99.87 % identity in the amino acid sequence) and GyrA (S83Y and D87A) (100 % coverage and 99.77 % identity in the amino acid sequence) identified in the ST37 historical isolates that were not mutated in contemporary isolates.

No mutations were identified in both contemporary and historical isolates in colistin and tigecycline resistance associated with MgrB, PmrB and RamR proteins (100 % coverage and 100 % identity in the amino acid sequence).

### Plasmids, integrative conjugative elements and phages in ST37

The three historical isolates harboured an IncF [F2:A-:B-] plasmid, carrying the *bla*_CTX-M-15_, *bla*_TEM-1_ and *bla*_OXA-1_ β-lactamase genes. In isolate SC26, IncF was simultaneously present within the same cell with an IncR plasmid, while in 9362 and 4011 strains small plasmids were detected (Table S1). Historical isolates carried a resistome coding for ESBLs, resistance to aminoglycosides, folate pathway inhibitors, chloramphenicol, macrolides and tetracyclines (Table 1). Remarkably the *armA* gene, already described in other ST37 isolates from 2014 [31], was detected in the 9362 genome located in the pKPN plasmid, indicating deletion of the entire transfer locus (Table 1, Fig. S1). Any of these plasmids and the relative resistome were detected in the contemporary isolates, but which acquired the pKpQIL plasmid carrying the *bla*_KPC_ gene. Beside pKpQIL, these strains carried the pKPN plasmid not harbouring any resistance gene (Table S1). The pKpQIL plasmid was not identified in the other 32 ST37 genomes retrieved from GenBank (Fig. 1).

**Fig. 2.**
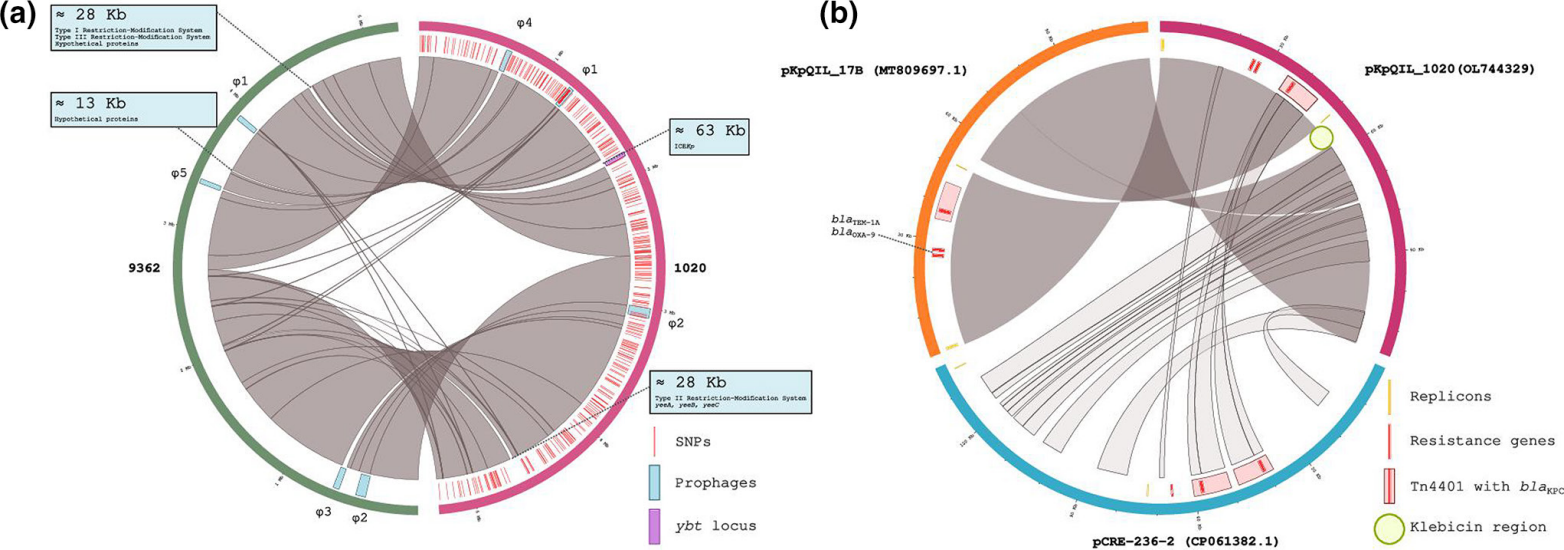
Synteny maps via Circos plots of 9362 and 1020 chromosomes and 1020, 17B and pCRE-236–2 plasmids comparing (**a**) the synteny between the chromosomal sequences of isolates 9362 and 1020 and (**b**) the synteny among the plasmid pKpQIL 1020 (from strain 1020, NCBI accession no. OL744329), 17B (a plasmid of an ST512 isolate from PUI hospital; MT809697) and the FIA plasmid, belonging to the phylogenetically closest ST37 isolate producing KPC-3 from Chicago (plasmid pCRE-236–2, CP061382).

In the chromosome, major genomic differences consisted of the presence or absence of prophages, restriction/modification systems (*yeeA, yeeB* and *yeeC* genes) and in the acquisition of the siderophore yersiniabactin (*ybt*) locus, which was located within the mobile genetic element ICE*Kp* [32] in contemporary isolates (Table S2). In these isolates the structural variant ICE*Kp*3, carrying the *ybt* lineage 9, integrated inside a tRNA-Asn located between the genes coding for a Na^+^/H^+^ antiporter and a nitrogen assimilation transcriptional regulator (Fig. 2a). The *ybt* ST414 was determined based on the combination of the alleles of the genes belonging to the *ybt* locus. To determine its diffusion in *K. pneumoniae* we performed a scan with Kleborate [23] on the 2934 completely assembled genomes downloaded from GenBank. Among these genomes, only the phylogenetically related ST37 strain from New York (ASM1417001) carried a similar *ybt* locus combination (single nucleotide variant, SNV, of *ybt* ST414).

Two prophages (φ1 and φ2 in Fig. 2a) were shared by all recent and old isolates from Rome, while prophages φ3 and φ5 were specific for the historical isolates, and φ4 for the contemporary isolates (Fig. 2a, Table S2). Neither the contemporary nor the historical isolates carried any CRISPR-Cas system.

These discontinuities between 9362 and 1020 genomes were confirmed by the Scoary tool [21] and by the analysis of the RAST [22] results (Table S2).

### Acquisition of *bla*_KPC_ in contemporary isolates

The contemporary ST37 isolates acquired *bla*_KPC_ on identical pKpQIL plasmids (100 % coverage and 99.99 or 100 % identity in the nucleotide sequence), with differences only in the *bla*_KPC_ genes, coding for the KPC variants KPC-31 (isolates 1016 and 1021), KPC-70 (isolate 1015) and KPC-110 (isolate 1020) (100 % coverage and 99.66 % identity in the amino acid sequence between KPC-31 and the KPC-70 and KPC-110 variants). Synteny analysis was performed among pKpQIL_1020 (from strain 1020, NCBI accession no. OL744329), pKpQIL_17B, a pKpQIL plasmid of an ST512 isolate from PUI (MT809697) and the FIA plasmid belonging to the phylogenetically closest ST37 isolate producing KPC-3 from Chicago (plasmid pCRE-236–2, CP061382) (Fig. 2b). The related strain from New York was positive for the *bla*_KPC-2_ gene located on a plasmid that had no similarity with those in Italian ST37.

The homology between pKpQIL_1020 and pCRE-2 was low, the two plasmids being significantly different in replicon content but both carrying the Tn4401 transposon with *bla*_KPC-3_-like genes. The pKpQIL_1020 and pKpQIL_17B plasmids, by contrast, were almost identical except for a 4.5 kb region, located between the IncFII *repA* replicase and the fertility inhibition *finO* genes. This 4.5 kb region, present in the ST37 plasmids but not in other pKpQIL plasmids circulating in PUI, coded for: klebicin B, a colicin immunity protein, a phospholipase D family protein and three hypothetical proteins. The presence of this specific region has been certified for the first time in KPC-producing *E. coli* carrying pKpQIL [33], but a GenBank search of the region revealed its presence in several *K. pneumoniae* isolates, belonging to multiple STs [34].

### The novel KPC-110 variant confers resistance to CZA in ST37

ST37 isolates from PUI acquired the pKpQIL plasmid carrying *bla*_KPC-3_-like genes. Specifically, two isolates (1016 and 1021) encoded the inhibitor-resistant KPC-31 [8]. Isolates 1015 and 1020 carried two different SNVs of the *bla*_KPC-31_ gene, coding for two variants named KPC-70 and KPC-110, respectively. Compared to KPC-3, KPC-70 displayed a T268A substitution in the 270-loop in addition to the D179Y substitution characteristic of KPC-31 located in the Ω-loop [35]. KPC-110, by contrast, displayed an G43R substitution alongside the D179Y one.

Variants of the KPC enzyme displaying only the D179Y substitution have been well described and documented, and the role of mutations inside the Ω-loop (amino acids 164–179 [35]) in the development of CZA resistance (paired with a restored susceptibility toward carbapenems) has been extensively reported [8, 9].

The KPC-70 variant was recently identified in an ST512 *K. pneumoniae* isolated in 2020 in PUI [10], and later reported in an ST13 *K. pneumoniae* from Portugal [36]. The KPC-110 variant was not previously identified.

Testing the MIC of CZA in TOP10 *E. coli* recipient cells expressing four KPC variants (KPC-3, KPC-31, KPC-70 and KPC-110) we noted how the strains producing KPC-70 and KPC-110 were more resistant to CZA (Table 2). In fact, in comparison with KPC-31, KPC-70 showed a twofold CZA MIC increase (from 24 to 48 mg l^−1^) and KPC-110 showed an MIC increase of at least tenfold (from 24 to >256 mg l^−1^). Increasing CZA MICs were also observed for the DH5α *E. coli* transformants carrying pKpQIL plasmids expressing KPC-70 and KPC-110 variants with respect to the transformant expressing KPC-31 (Table 2).

**Table 2.** MICs of *E. coli* pKpQIL transformants and recombinant clones carrying *bla*_KPC_ gene variants cloned in pTOPO vector from *K. pneumoniae* ST37 strains isolated in 2021, Rome, Italy

Isolate	KPC-variant	Mutations compared to KPC-3	CZA MIC (mg l^−1^)	MEM MIC (mg l^−1^)
1020 KP	110 (New)	G43R, D179Y	128	<0.12
1020 pKpQIL			48	<0.12
1020 Topo			>256	<0.12
1015 KP	70	D179Y, T268A	>256	<0.12
1015 pKpQIL			12	<0.12
1015 Topo			48	<0.12
1016 KP	31	D179Y	48	<0.12
1016 pKpQIL			8	<0.12
1016 Topo			24	<0.12
1021 KP	31	D179Y	16	<0.12
1021 pKpQIL			8	<0.12
1021 Topo			24	<0.12
3 KP	3	neg	0.75	16
3 pKpQIL			0.75	1
3 Topo			1	16
DH5-α	neg	neg	0.125	<0.12
Top10	neg	neg	0.125	<0.12

KP, *Klebsiella pneumoniae *; Topo, construct cloned in pTOPO vector.

## Conclusions

The ST37 clone was widely represented in the Italian epidemiology before the spread of the *bla*_KPC_-harbouring isolates belonging to CG258. Several descriptions of ST37 in hospitals of Rome were made in 2010 [5, 7, 26].

To the best of our knowledge, between 2011 and 2021, there were very few ST37 isolates described in Italian hospitals. In that decade, the Italian epidemiology was characterized by carbapenem-resistant *K. pneumoniae* isolates belonging to ST307, ST258 and ST512 [37–40]. The presence of *bla*_KPC-3_ in ST37 was reported only in one isolate from the centre of Italy in the nationwide collection of carbapenem-resistant invasive isolates, obtained in 2016 in the framework of the National Antibiotic-Resistance Surveillance (AR-ISS) [2]. Additionally, the number of complete *K. pneumoniae* ST37 genomes available in the GenBank database is very limited, suggesting narrow diffusion of this clone worldwide.

Given this context, we hypothesize that the ST37 clone was present in PUI for over a decade, probably within the wider group of carbapenem-susceptible *K. pneumoniae* strains. It was subsequently reported by the surveillance system when it shifted in the CZA-resistant pool by acquisition of the *bla*_KPC_ gene variants. We consider that the historical ertapenem-resistant strains from 2010 [5] were not the direct ancestors of the KPC-producing CZA resistant strains from 2021.

It is fascinating to observe how the genomic analysis highlighted the evolutionary pathways of these isolates, including the acquisition of ICE*Kp* harbouring the *ybt* loci, the differences in phage content and the gain of *bla*_KPC_ harbouring the pKpQIL plasmid.

It has recently been demonstrated that the wild-type OmpK36 porin is relevant in stabilizing the mating pair during conjugation of the pKpQIL plasmid. Mating pair stabilization occurs by interaction between OmpK36 and the TraN factor of pKpQIL [41]. In ertapenem-resistant isolates 4011 and SC26, the insertion at the glycine–aspartic acid site, observed in the OmpK36 L3 loop, could have limited the acquisition by conjugation of pKpQIL in these strains. This could explain why ST37 from the 2010s, which was a highly diffused clone at PUI, did not participate significantly in the acquisition of *bla*_KPC_ on pKpQIL. Instead, the introduction and spread of CG258 occurred intensively in the hospital [5, 7]. The acquisition of pKpQIL-*bla*_KPC_ happened in more recent times, starting from a susceptible ST37 strain, carrying a wild-type OmpK36.

Interestingly, ST37 could have acquired the pKpQIL encoding KPC-31, and during the outbreak it led to two further variants in a month. The KPC-70 and KPC-110 variants, compared to KPC-31, showed an increase in CZA MIC. This implies that ST37 has high mutability capacity, as also suggested by the high numbers of SNPs identified between historical and contemporary strains. Because of the increasing use of CZA for treating carbapenem-resistant *K. pneumoniae* infections, it can be expected that ST37 will spread in the hospital in the near future.

## Supplementary Data

Supplementary material 1

Supplementary material 2
