# Supplementary material for: Ceftazidime–avibactam resistance in Klebsiella pneumoniae sequence type 37: a decade of persistence and concealed evolution
Source: Microb Genom. 2023 Feb 8;9(2):mgen000931. doi: 10.1099/mgen.0.000931 (PMC9997735; doi:10.1099/mgen.0.000931)
Supplement: Supplementary material 1 [file mgen-9-931-s001.pdf]

Strain	Chromosome	ORFs	Plasmids-Replicons-Resistance content				Accession Nos.
			pKpQIL	pKPN	IncFII	Other	
1020	5,264,949 bp	5110	116,484 bp FIIk-FIB(pQil) *bla*$_{KPC-110}$, *bla*$_{OXA-9}$, *bla*$_{TEM-1A}$	112,694 bp FIB(K)	neg	3,302 (ColpHAD28)	OL744329 (pKpQIL)
1021	5,265,397 bp	5104	116,484 bp FIIk-FIB(pQil) *bla*$_{KPC-31}$, *bla*$_{OXA-9}$, *bla*$_{TEM-1A}$	112,694 bp FIB(K)	neg	3,302 (ColpHAD28)	
9362	5,250927 bp	5155	neg	122,004 bp FIB(K) *armA*, *bla*$_{TEM-1B}$, *catA1*, *dfrA5*, *sul1*, *mph*(E), *msr*(E), *tet*(D)	79,897 bp FII *bla*$_{CTX-M-15}$, *bla*$_{OXA-1}$, catB4, *aac3-IIa*, *aac(6')-Ib*	109,627 bp (phage) 9,294 bp (ColRNAI) 5,207 (Col440II)	
4011	5,254,010 bp	5180	neg	177,516 bp FIB(K) *catA1*, *dfrA5*, *sul1*	79,764 bp FII *bla*$_{CTX-M-15}$, *bla*$_{OXA-1}$, catB4, *aac3-IIa*, *aac(6')-Ib*	109,627 bp (phage) 9,294 bp (ColRNAI) 7,832 (Col440I), 5,207 (Col440II)	

Table S1. Characteristics and features of the completely assembled ST37 genomes

Features	Positions Accession No.	1020	1021	9362	4011
Prophage ϕ 3	592,872-634,630 (9362)	-	-	+	+
Prophage ϕ 4	659,466-693,924 (1020)	+	+	-	-
Prophage ϕ 5	659,663-673,038 (9362)	-	-	+	+
Hypothetical proteins-integrase	831,536-845,096 (9362)	-	-	+	+
Type III restriction-modification system Type I restriction enzyme EcoR124II M	1,768,009-1,795,762 (9362)	-	-	+	+
ICE*Kp*[a]	1,780,831-1,845,323 (1020)	+	+	-	-
Pseudo-prophage P4	4,615,564-4,625,017 (9362)	-	-	+	+
Type II restriction enzyme, methylase subunit YeeABC	4,612,089-4,638,257 (1020)	+	+	-	-
Two-component system response regulator PgtA GrpB family protein aldo/keto reductases	2,562,989-2,570,329 (4011)	+	+	-	+

[a]: Integrative Conjugative Element *Klebsiella pneumoniae* ref

Table S2. Major chromosomal differences identified by WGS comparison among ST37 genomes

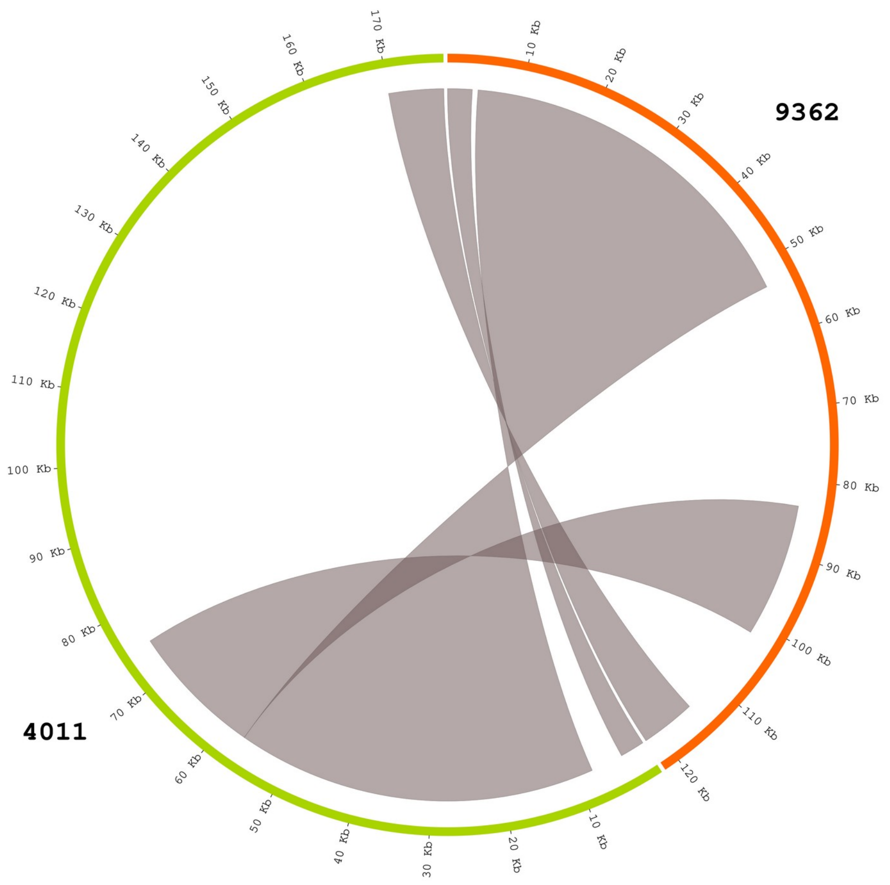

Supplementary Figure SF1: Circos plot comparing the synteny between the pKPN plasmid sequences of the historical isolates 9362 (in orange) and 4011 (in green)
